# Knockdown lncRNA NEAT1 regulates the activation of microglia and reduces AKT signaling and neuronal apoptosis after cerebral ischemic reperfusion

**DOI:** 10.1038/s41598-020-71411-1

**Published:** 2020-11-12

**Authors:** Xunran Ni, Qian Su, Wenbo Xia, Yanli Zhang, Kejuan Jia, Zhiqiang Su, Guozhong Li

**Affiliations:** 1 The First Affiliated Hospital of Harbin Medical University, Harbin, 150001 People’s Republic of China; 2 The Key Laboratory of Myocardial Ischemia, Harbin Medical University, Ministry of Education, Harbin, People’s Republic of China

**Keywords:** Immunology, Neuroscience, Diseases, Neurology

## Abstract

Acute cerebral ischaemia may lead to serious consequences, including brain injury caused by uncontrolled reperfusion, which occurs when circulation is re-established. The long non-coding RNA (lncRNA) nuclear enriched abundant transcript 1 (NEAT1) plays an important role in the immune system. However, the potential roles and underlying molecular mechanisms of NEAT1 in cerebral ischaemia/reperfusion (I/R) injury remain unclear. The aim of the present study was to investigate the function of the lncRNA NEAT1 in cerebral I/R injury and its potential beneficial effects on neurons. In our study, oxygen–glucose deprivation (OGD)/reoxygenation (OGD/R) was induced in vitro to mimic cerebral I/R injury. Cholecystokinin-octopeptide (CCK-8) was used to measure cell viability, and flow cytometry was used to measure cell apoptosis. Real-time quantitative PCR (qRT-PCR) was used to measure the expression of phenotypic markers of classically activated (M1) and alternatively activated (M2) microglia, and western blotting was performed to detect the levels of proteins related to the AKT/STAT3 pathway. The expression of the lncRNA NEAT1 was significantly upregulated in patients with ischaemic stroke, and knockdown of the lncRNA NEAT1 alleviated OGD/R-induced apoptosis and increased neuronal viability. Furthermore, the lncRNA NEAT1 may inhibit microglial polarization towards the M1 phenotype to reduce the damage caused by OGD/R and reduce the activity of the AKT/STAT3 pathway. In conclusion, the lncRNA NEAT1 may be a potential target for new therapeutic interventions for cerebral I/R.

## Introduction

Cerebrovascular diseases, which have high disability and mortality rates, are becoming increasingly common in the elderly^1^. The only method for preventing permanent cerebral ischaemia-induced damage is to restore blood flow to the ischaemic region of the brain as quickly as possible. Nevertheless, severe damage, called ischaemia/reperfusion (I/R) injury, occurs after artery recanalization^2^. The main pathophysiological characteristics of I/R injury include an immune response and apoptosis^3^. However, specific drugs for treating I/R injury are not available in the clinic due to the lack of understanding of its mechanisms^4^.

Long non-coding RNAs (lncRNAs) are RNAs longer than 200 nucleotides that do not encode proteins^5^. In recent years, lncRNAs have been shown to participate in various pathological and physiological processes, such as apoptosis, cell cycle progression, differentiation and inflammation^6^. The lncRNA nuclear enriched abundant transcript 1 (NEAT1) has been reported to be involved in various diseases^7,8^, has been proven to be related to myocardial I/R injury^9^, and has been suggested to promote the activation of the inflammasome in macrophages^10^. Interestingly, NEAT1 was recently shown to be transcriptionally activated by Yin Yang 1 (YY1) and to contribute to the oxygen–glucose deprivation (OGD)/reoxygenation (OGD/R) injury of microglial cells by activating the Wnt/β-catenin signalling pathway^11^.

Microglia are the main immune cells of the central nervous system (CNS) and exert diverse functions in the pathogenesis of various neurological diseases^12^. Once activated, microglia serve as a double-edged sword in the pathological processes of CNS diseases^13^. During acute brain injury, classically activated (M1) microglia release some cytokines and exacerbate inflammatory damage^13^. Alternatively activated (M2) microglia exert neuroprotective effects through phagocytosis and the removal of cell debris, the attenuation of local inflammation, and by participating in tissue remodelling^14^.

Hence, in this experiment, we hypothesized that the lncRNA NEAT1 regulates microglial activation and promotes neuronal apoptosis after cerebral I/R injury. This study provides a potential target for new therapeutic interventions for cerebral I/R injury.

## Method

### Patients and blood samples

Blood specimens and clinical data were obtained from patients with acute ischaemic stroke (AIS) at the Department of Neurology, The First Affiliated Hospital of Harbin Medical University between 2019 and 2020. Blood specimens from normal subjects were obtained from the Physical Examination Center, The First Affiliated Hospital of Harbin Medical University. The research was performed in accordance with relevant regulations and informed consent was obtained from all participants. The protocol was approved by the Institutional Research Ethics Committee of The First Affiliated Hospital of Harbin Medical University.

### Cell culture

BV-2 cells were maintained in 1 × alpha minimum essential medium with Eagle’s salts, ribonucleosides, deoxyribonucleosides & L-glutamine (MEM, Corning, NY, USA) supplemented with 10% foetal bovine serum (FBS, HyClone, LA, USA), 1% sodium pyruvate (SP, 100 mmol, Gibco, CA, USA) and 1% penicillin/streptomycin (PS; 100 IU/ml penicillin and 100 µg/ml streptomycin). N2a cells were maintained in Dulbecco’s Modified Eagle’s Medium (DMEM; Corning) supplemented with 10% FBS (HyClone) and 1% penicillin/streptomycin (PS; 100 IU/ml penicillin and 100 µg/ml streptomycin). The cells were cultured in an incubator with 5% CO_2_ at 37 °C.

### Preparation of the lentiviral vector and infection

Lentiviruses expressing the NEAT1 siRNA (si-NEAT1) or a control siRNA (si-con) were packaged by HanBio (Shanghai, China). The packaged recombinant lentiviruses were transfected into BV-2 cells to knockdown NEAT1, and puromycin was applied for selection for at least 15 days (Supplementary information).

### OGD/R

OGD was induced in microglia and neurons as described previously^15^. Briefly, the original culture medium was first removed and replaced with glucose/serum-free DMEM. Then, the plates were transferred to an anaerobic chamber with an atmosphere containing 5% CO_2_ and 95% N_2_ without oxygen for 2 h at 37 °C. The BV-2 cells were returned to normoxic conditions with regular medium to terminate OGD and initiate reperfusion. The N2a cells were returened to normoxic conditions with different supernatant of OGD/R treatment BV-2 cells to terminate OGD and initiate reperfusion.

### Quantitative real-time PCR (qRT-PCR)

Total RNA was extracted from cells using TRIzol (Life Technologies Carlsbad, CA, USA) and from blood using TRIzol LS Reagent (Life Technologies Carlsbad). The cDNA templates were prepared in a 10-µl reaction using the ReverTra Ace qPCR RT Kit (TOYOBO CO., Osaka, Japan), and real-time PCR analyses were performed in triplicate using FastStart Universal SYBR Green Master (Rox) (Roche, Mannheim, Germany). All qRT-PCR experiments and data analysis procedures were performed in accordance with The Minimum Information for Publication of Quantitative Real-Time PCR Experiments (MIQE) guidelines.

### Protein isolation and western blot analysis

Cells were lysed on ice in RIPA lysis buffer containing protease inhibitors and phosphatase inhibitors (Beyotime, China) via sonication for two 10-s pulses with 30 s between pulses. The cell lysates were cleared by centrifugation at 12,000 rpm for 10 min at 4 °C, and the supernatant was retained as the total protein. Proteins were quantified using a bicinchoninic acid (BCA) assay kit (Sigma Aldrich, USA). We performed 10% sodium dodecyl sulfate–polyacrylamide gel electrophoresis (SDS-PAGE) to separate the proteins. The proteins were then transferred to a nitrocellulose membrane through electroblotting. The membrane was blocked with a 5% blocking solution (non-fat milk) and then incubated overnight at 4 °C with blocking buffer containing the following primary antibodies: AKT, STAT3, P-AKT, P-STAT3, and β-actin. On the next day, a horseradish peroxidase (HRP)-conjugated goat secondary antibody was added to the blocking buffer (1:1,000, non-fat milk), and a chemiluminescence kit (Thermo Fisher Scientific, USA) was used to detect the proteins. The Tanon Gel Image System (Tanon Science & Technology Co., Ltd.) was used for photography and quantification.

### Cholecystokinin-octopeptide (CCK-8) assay

CCK-8 was used to verify whether the lncRNA NEAT1 affects neuronal viability. N2a cells were exposed to OGD as previously described. After OGD exposure, N2a cells were cultured with the supernatant of OGD/R-exposed lncRNA NEAT1 knockdown BV-2 cells or untreated lncRNA NEAT1 knockdown BV-2 cells and cultured for 24 h. Then, 10 μl of CCK-8 reagent (Beyotime, China) were added to each well, and the cells were incubated for 2 h at 37 °C. A microplate reader (Tecan, Switzerland) was employed to detect the absorbance at 450 nm.

### Flow cytometry

Flow cytometry was utilized to detect the apoptosis of neuronal cells. N2a cells were treated as described above and then collected and resuspended in binding buffer. The FITC-Annexin V Apoptosis Detection Kit I (BD Pharmingen, USA) was used to stain the cells with FITC-Annexin V and propidium iodide (PI). A BD FACS CantoII flow cytometer was used to analyse the apoptotic cells.

### Statistical analysis

All data were statistically analysed with GraphPad Prism 7.0 software (GraphPad Software Inc., San Diego, CA, USA). Statistical comparisons were performed using two-tailed Student t-test for two groups and one-way ANOVA with Dunnett’s multiple comparison test for more than three groups. Asterisks are used to indicate statistical significance: *, **, ***, and **** indicate p < 0.05, p < 0.01, p < 0.001, and p < 0.0001, respectively.

## Results

### The lncRNA NEAT1 is upregulated in cerebral ischaemia

**Figure 1:**
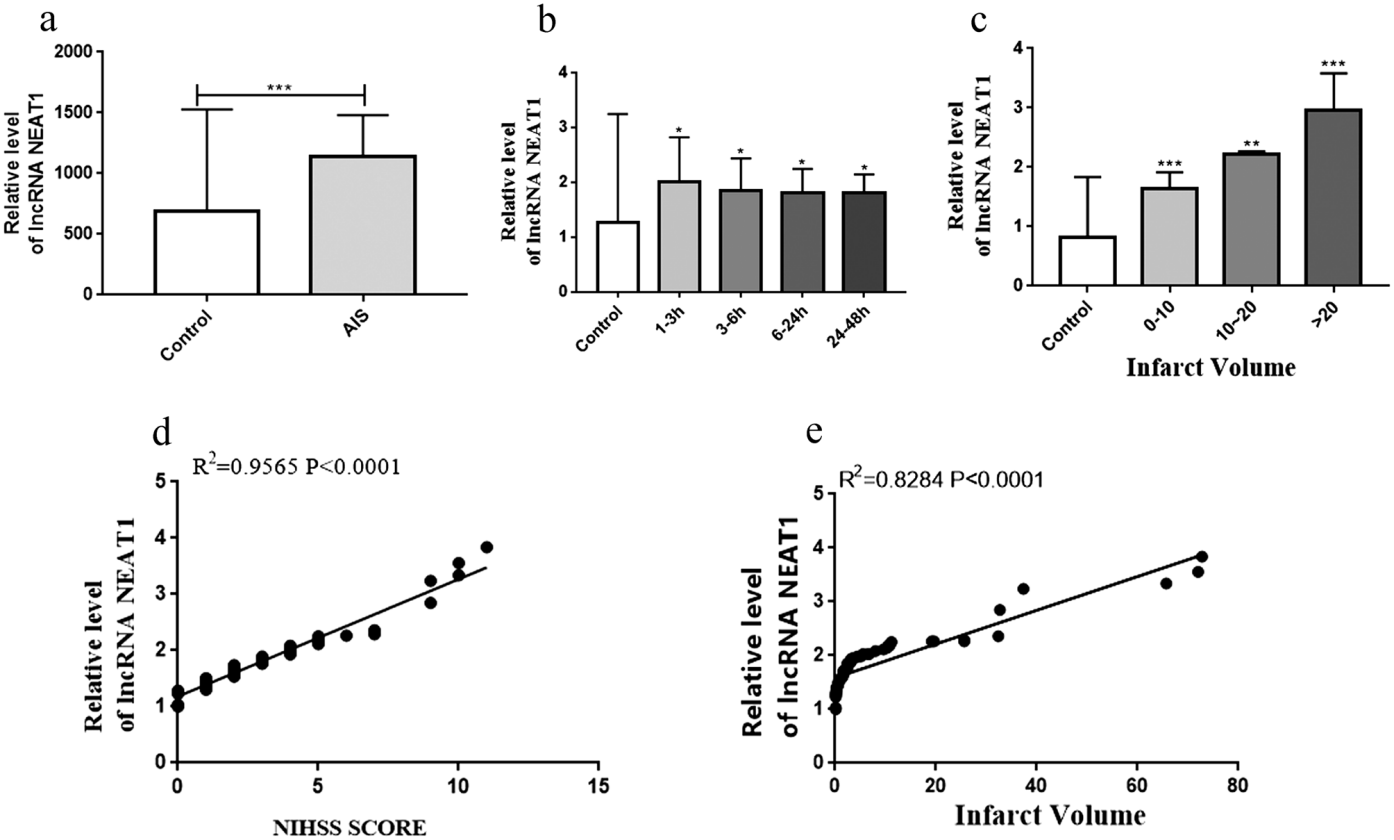
NEAT1 was upregulated in cerebral ischaemia. **(a)** Expression of NEAT1 in the blood of AIS patients (n = 64) and healthy controls (n = 64) detected by qRT-PCR. **(b)** Expression of NEAT1 in patients with different onset times compared with healthy controls. **(c)** Expression of NEAT1 in patients with different infarct volumes compared with healthy controls. **(d)** Linear regression analysis of NEAT1 expression and NIHSS scores was conducted for each individual. **(e)** Linear regression analysis of Neat1 expression and infarct volume was conducted. Asterisks mark signifcant diferences(*, **, ***, and**** indicate p < 0.05, p < 0.01, p < 0.001, and p < 0.0001, respectively).

qRT‐PCR was used to determine the relative expression of NEAT1 and investigate whether NEAT1 is associated with cerebral ischaemic stroke in patients with AIS. The basic characteristics of the participants are provided in Table 1. In our study, NEAT1 was expressed at markedly higher levels in the blood of patients with AIS than in controls (Fig. 1a). In addition, NEAT1 expression was positively correlated with the time of onset of illness (Fig. 1b) and the cerebral infarct volume (Fig. 1c). Furthermore, NEAT1 expression was positively correlated with stroke severity, which was evaluated based on National Institute of Health Stroke Scale (NIHSS) scores (Fig. 1d) and infarct volume (Fig. 1e).

### OGD/R exposure and preliminary analysis of lncRNA NEAT1 expression

**Figure 2:**
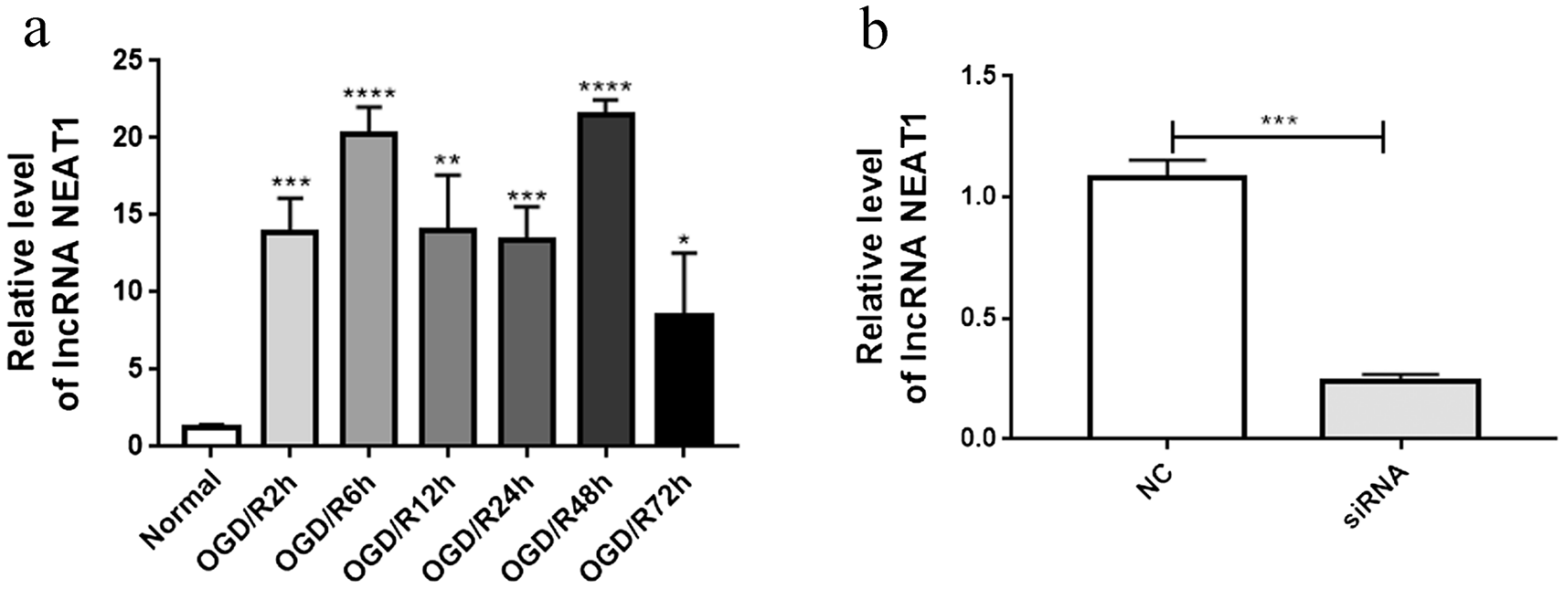
Preliminary analysis of lncRNA NEAT1. **(a)** The expression of lncRNA NEAT1 was increased after exposure to OGD/R. **(b)** Knockdown of lncRNA NEAT1 was verified via qRT-PCR. The asterisks indicate significant differences. Asterisks mark signifcant diferences(*, **, ***, and**** indicate p < 0.05, p < 0.01, p < 0.001, and p < 0.0001, respectively).

BV-2 cells were cultured in serum-free and glucose-free medium to mimic cerebral I/R injury. After exposure to OGD for 2 h, BV-2 cells underwent reperfusion for 2, 6, 12, 24, 48, or 72 h. Then, we detected the expression of the lncRNA NEAT1 in BV-2 cells. The expression of the lncRNA NEAT1 was significantly increased after OGD/R exposure and reached the highest levels at 48 h of reperfusion (Fig. 2a); thus, we used 2 h of OGD and 48 h of reperfusion for subsequent experiments. We used an siRNA packaged in a lentiviral vector to knockdown the lncRNA NEAT1. As shown in Fig. 2b, the expression of the lncRNA NEAT1 was downregulated in NEAT1 knockdown BV-2 cells compared to si-con-treated BV-2 cells. After si-NEAT1-treated BV-2 cells and si-con-treated cells were exposed to OGD for 2 h and reperfusion for 48 h, we collected the cells, extracted RNA and protein, and collected the cell supernatant for use with N2a cells after exposure to OGD.

### Effect of the lncRNA NEAT1 on neurons subjected to cerebral I/R injury

**Figure 3:**
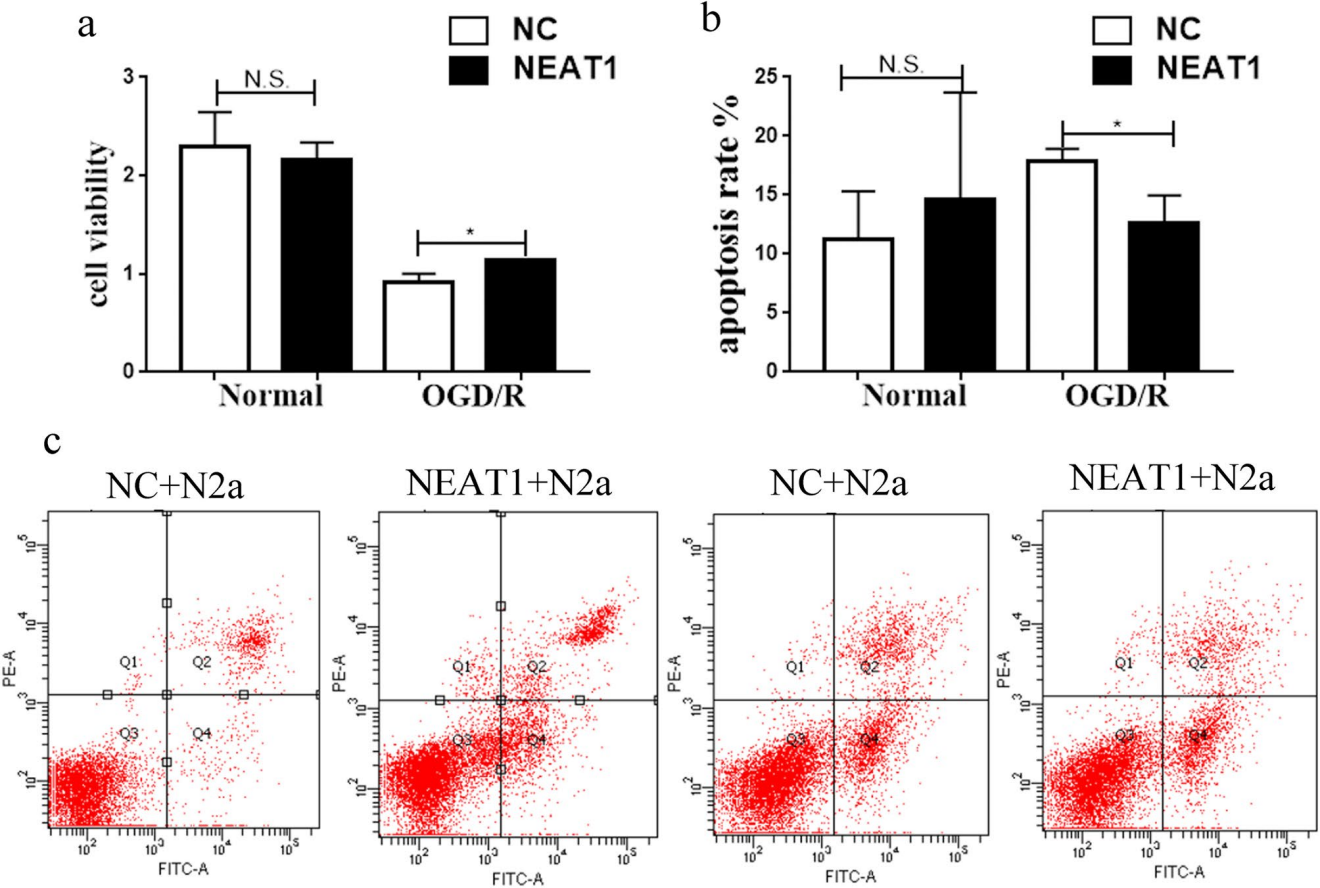
Function of lncRNA NEAT1 in cerebral I/R injury. **(a)** The cell viability assay revealed that cell viability was higher in the lncRNA NEAT1 knockdown group than the untreated group. **(b)**, **(c)** Flow cytometry revealed that apoptosis was lower in the lncRNA NEAT1 knockdown group compared with the untreated group. Asterisks mark signifcant diferences. Asterisks mark signifcant diferences(*, **, ***, and**** indicate p < 0.05, p < 0.01, p < 0.001, and p < 0.0001, respectively).

N2a cells were cultured with the supernatant of OGD/R-treated lncRNA NEAT1 knockdown cells, control BV-2 cells or untreated BV-2 cells for 24 h after OGD exposure to investigate the function of the lncRNA NEAT1 in cerebral I/R injury. Then, CCK-8 was used to detect neuronal viability. The CCK-8 assay revealed statistically significant differences in the viability of N2a cells cultured with the supernatant of the two groups of OGD/R-exposed cells compared with N2a cells cultured with the supernatant of untreated cells (Fig. 3c). We also performed flow cytometry to detect apoptosis, and a significant difference was observed in the apoptosis of N2a cells cultured with the supernatants of cells from the two OGD/R-exposed groups compared with N2a cells cultured with the supernatant of untreated cells (Fig. 3a, b). Thus, knockdown of the lncRNA NEAT1 protects N2a cells from cerebral I/R injury and the lncRNA NEAT1 possesses an anti-apoptotic effect.

### The lncRNA NEAT1 inhibits microglial polarization towards the pro-inflammatory (M1) phenotype

**Figure 4:**
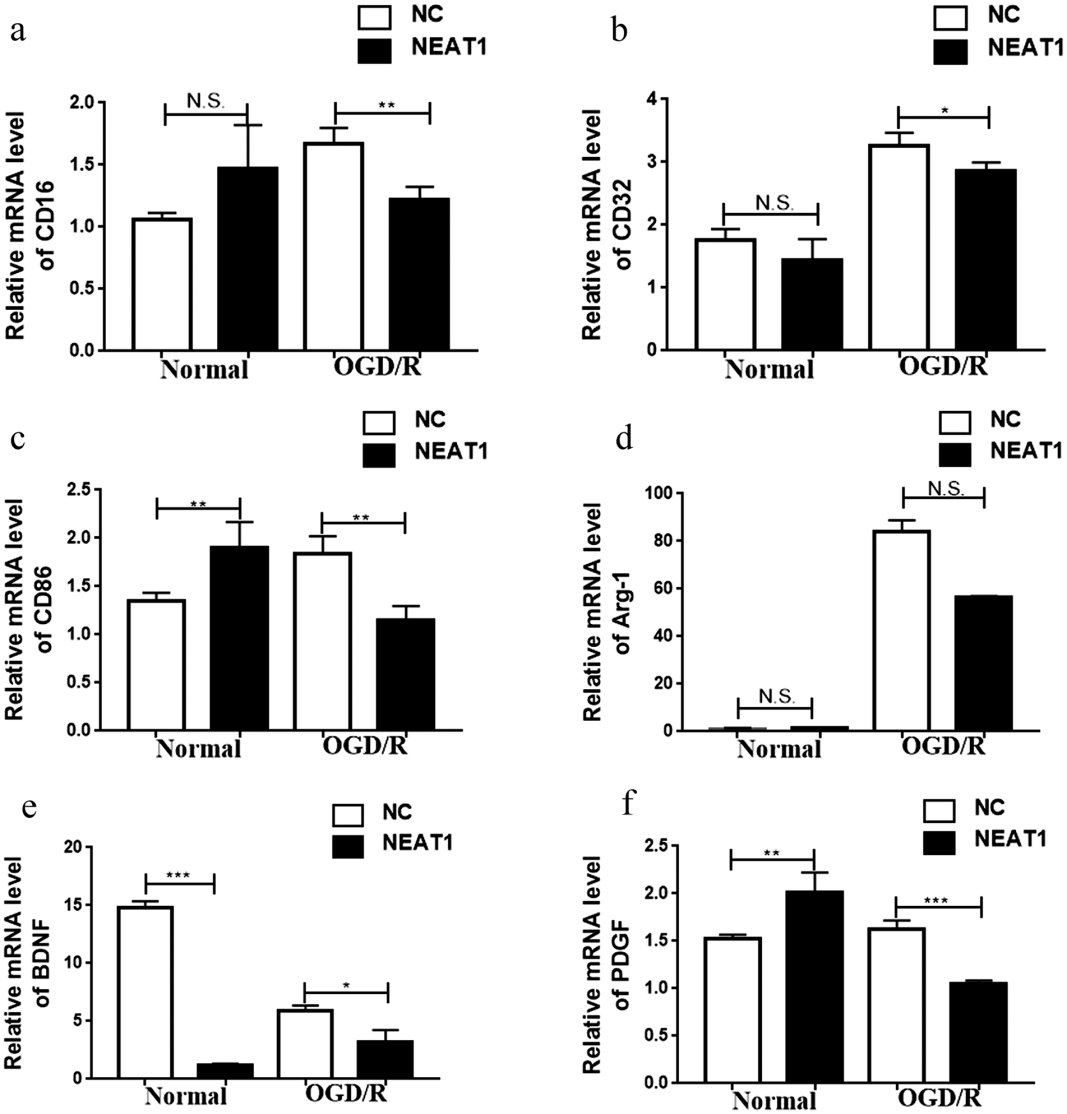
Function of lncRNA NEAT1 of microglial polarization. Determined by qRT-PCR, the mRNA expression levels of M1 markers **(a)** CD16, **(b)** CD32, **(c)** CD86 and M2 markers **(d)** Arg-1, **(e)** BDNF, **(f)** PDFG indicated that NEAT1 deficiency suppresses M1 microglial polarization but does not promote M2 microglial polarization in OGD/R-exposed microglial cells. Asterisks mark signifcant diferences(*, **, ***, and**** indicate p < 0.05, p < 0.01, p < 0.001, and p < 0.0001, respectively).

We exposed NEAT1 knockdown BV-2 cells to OGD/R and then detected the mRNA expression of markers of M1 and M2 microglia. Prior to the OGD/R treatment, the expression of CD16 and CD32 was not significantly different between NEAT1 knockdown cells and control cells, and CD86 was expressed at higher levels in NEAT1 knockdown cells than in control cells. After the OGD/R treatment, the M1 markers CD16, CD32, and CD86 were expressed at lower levels in the NEAT1 knockdown cells than in control cells (Fig. 4a–c). The expression of M2 markers (BDNF, PDGF, and Arg-1) was also analysed. Prior to the OGD/R treatment, the expression of Arg-1 was not significantly different between NEAT1 knockdown cells and control cells, BDNF was expressed at lower levels in NEAT1 knockdown cells than in control cells, and PDGF was expressed at higher levels in NEAT1 knockdown cells than in control cells. After the OGD/R treatment, a significant difference in the expression of Arg-1 was not observed between NEAT1 knockdown cells and control cells, BDNF was still expressed at lower levels in NEAT1 knockdown cells than in control cells, and PDGF was expressed at lower levels in NEAT1 knockdown cells than in control cells (Fig. 4d–f). Based on these data, NEAT1 suppresses M1 microglial polarization but does not promote M2 microglial polarization in OGD/R-exposed microglial cells.

### The lncRNA NEAT1 reduces the activity of the AKT/STAT3 pathway

**Figure 5:**
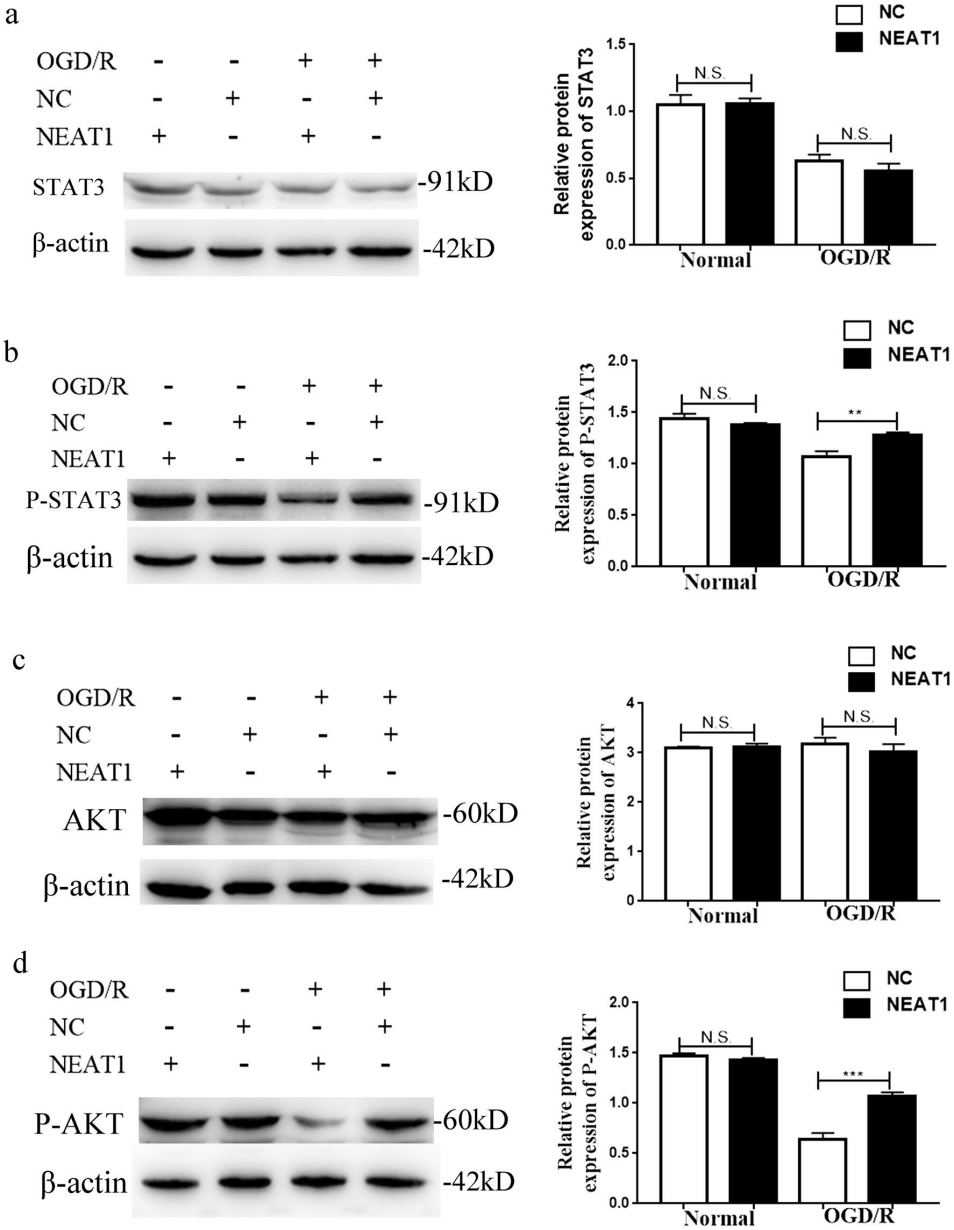
Quantification of STAT3 **(a)**, P-STAT3 **(b)**, AKT **(c)** and P-AKT **(d)** protein expression in cells from the four groups. β-Actin was used as a loading control. The signals were normalized and quantified with Tanon software. Asterisks mark signifcant diferences(*, **, ***, and**** indicate p < 0.05, p < 0.01, p < 0.001, and p < 0.0001, respectively).

Both AKT and STAT3 play important roles in many physiological processes. The AKT/STAT3 pathway was analysed using western blotting. As shown in Fig. 5a, c, the levels of the total STAT3 and AKT proteins were unchanged. Moreover,the phosphorylation of both AKT and STAT3 was reduced in the NEAT1 knockdown BV-2 cells compared with the control cells after OGD/R (Fig. 5b, d). Thus, the activities of both AKT and STAT3 are suppressed by the lncRNA NEAT1 in BV-2 cells cultured under OGD/R conditions.

## Discussion

Recently, numerous lncRNAs have been shown to participate in cerebral I/R injury^16^. Hence, the identification of prognostic lncRNAs for cerebral I/R injury is necessary and important. In addition, Han and Zhou have already confirmed that NEAT1 contributes to OGD/R injury in microglial cells^11^, which provides a theoretical basis for our research. In the present study, we used OGD to mimic ischaemic stroke conditions in vitro in BV-2 and N2a cells and to explore the potential mechanism of cerebral I/R injury. NEAT1 expression was upregulated in BV-2 cells following OGD/R when reperfusion lasted for 48 h, indicating that the lncRNA NEAT1 might play an important role in cerebral I/R injury. In addition, qRT-PCR, western blotting, flow cytometry and a cell viability assay were performed to evaluate the function of the lncRNA NEAT1.

I/R may cause apoptosis, autophagy, necrosis and necroptosis^17–19^. Severe I/R injury induces cell death via apoptotic or necrotic pathways^20^. As shown in the present study, knockdown of the lncRNA NEAT1 alleviated OGD/R-induced N2a cell apoptosis and increased N2a cell viability.

The AHA/ASA guidelines recommend early revascularization for AIS^21^. Moreover, preserving the function of nerves and organs following I/R injury is a challenge for physicians^22^. The most important strategies employed following cerebral I/R injury are controlling cell damage and protecting organ function^23^. The mechanisms of I/R injury are extremely complicated and diverse, and the immune response may be one of the most important mechanisms. When the blood supply is re-established after ischaemia, inflammation increases^15^. Therefore, approaches targeting immune activation represent an emerging therapeutic strategy for the treatment of I/R injury^22^. Microglia are important in the CNS and regulate the immune and inflammatory responses in the brain following ischaemic injury^23^. Microglia commonly exhibit a neural-specific phenotype and retain a relatively quiescent surveillance phenotype to constantly monitor the brain parenchyma^24–26^. During the process of neurological inflammation, microglial cells perform different functions through polarization towards different phenotypes^27^. In our study, the lncRNA NEAT1 may have inhibited microglial polarization towards the pro-inflammatory (M1) phenotype; however, the detailed mechanism by which the lncRNA NEAT1 controls microglia M1 is not clear and requires further study.

AKT, also known as protein kinase B (PKB), is an important factor regulating cell death and growth^28,29^. According to recent evidence, AKT activates STAT3, as it acts upstream of STAT3^30^, and STAT3 plays an important role in proliferation and astrogliosis^31^. AKT/STAT3 are also part of the IL-6 pathway, which plays a crucial role in immune reactions and may be involved in cerebral I/R injury. Notably, according to our western blot analysis, the phosphorylation of both AKT and STAT3 was reduced in OGD/R-exposed NEAT1 knockdown cells compared to OGD/R-exposed control cells; nevertheless, the levels of the total AKT and STAT3 proteins remained unchanged. Changes in the levels of the phosphorylated and total AKT and STAT3 proteins were not observed between OGDR-exposed NEAT1 knockdown cells and OGD/R-exposed control cells. Thus, the association between NEAT1 and the AKT/STAT3 pathway may not involve the direct regulation of signalling molecule expression by NEAT1.

Microglia, which act as macrophages in the CNS, have the same functions as mononuclear phagocytes^31^. However, microglia are more restricted than macrophages in their capacity to adopt an M2 phenotype and cytokine profile^32^. Both macrophages and microglia showed greater induction of gene expression in response to M1 polarization than M2 polarization^31^. These findings might explain why the microglia M2 phenotype was not significantly altered in our study.

In summary, the lncRNA NEAT1 exerts a neuroprotective effect, significantly inhibits the polarization of microglia to the M1 phenotype and reduces the activity of the AKT/STAT3 pathway. However, we did not clearly determine whether NEAT1 promotes the polarization of microglia towards the M2 phenotype, and the detailed mechanism by which NEAT1 controls microglial cell polarization is unknown and requires further study.

## Conclusions

The expression of the lncRNA NEAT1 is upregulated after cerebral ischaemic injury. Cerebral I/R may cause more severe damage than cerebral ischaemia. Knockdown of the lncRNA NEAT1 significantly reduces microglial polarization towards the pro-inflammatory (M1) phenotype and cell apoptosis and increases the activity of neuronal cells after OGD/R exposure. Furthermore, knockdown of the lncRNA NEAT1 inhibits the activity of the pro-inflammatory AKT/STAT3 pathway. These findings may provide new strategies for the treatment of cerebral I/R from an epigenetic perspective.

### Clinical perspectives

Cerebral I/R injury may cause more severe symptoms than ischaemic stroke in the clinic. However, the biological mechanism of cerebral I/R injury is not completely understood.

In our study, the lncRNA NEAT1 was expressed at higher levels in patients with ischaemic stroke than in normal subjects and was related to the cerebral infarct volume. In addition, knockdown of the lncRNA NEAT1 reduced neuronal apoptosis and inhibited microglial polarization towards the pro-inflammatory (M1) phenotype.

The lncRNA NEAT1 may be a potential treatment target for cerebral I/R, and our research may provide evidence for a new clinical strategy.

## Supplementary information

Supplementary Information.

## References

[1] Feng, X. *et al*. Estrogen and propofol combination therapy inhibits endoplasmic reticulum stress and remarkably attenuates cerebral ischemia-reperfusion injury and OGD injury in hippocampus. *J. Biomed. Pharmacother.* **108**, 1596–1606. 10.1016/j.biopha.2018.09.167 (2018) 10.1016/j.biopha.2018.09.167 30372862

[2] Feng, Y. *et al*. Tetrahydroxystilbene glucoside suppresses NAPDH oxidative stress to mitigate apoptosis and autophagy induced by cerebral ischemia/reperfusion injury in mice. *J. Evid.-Based Complement. Altern. Med.* **2019**(3913981), 1–9, 10.1155/2019/3913981 (2019) 10.1155/2019/3913981 PMC6662418 31379960

[3] Wu, M.Y. *et al*. Current mechanistic concepts in ischemia and reperfusion injury. *J. Cell. Physiol. Biochem.* **46**(4), 10.1159/000489241 (2018) 10.1159/000489241 29694958

[4] Dai, Q. L. *et al*. Berberine protects against ischemia-reperfusion injury: a review of evidence from animal models and clinical studies. *J. Pharmacol. Res.* **10**, 148, 10.1016/j.phrs.2019.104385 (2019) 10.1016/j.phrs.2019.104385 31400402

[5] Mercer, T. R., Dinger, M.E. & Mattick, J.S. Long non-coding RNAs: Insights into functions. *J. Nat. Rev. Genet.* **10**(3), 155–159, 10.1038/nrg2521 (2009) 10.1038/nrg2521 19188922

[6] Gutschner, T. & Diederichs, S. The hallmarks of cancer: A long non‐coding RNA point of view. *J. RNA Biol.* 9:703–719. 10.4161/rna.20481 (2012) 10.4161/rna.20481 PMC3495743 22664915

[7] Imamura K (2014). Long noncoding RNA NEAT1-dependent SFPQ relocation from promoter region to paraspeckle mediates IL8 expression upon immune stimuli. J. Mol Cell..

[8] Chen, Y. *et al*. Long non-coding RNA NEAT1 plays an important role in sepsis-induced acute kidney injury by targeting miR-204 and modulating the NF-κB pathway. *J. Int. Immunopharmacol.* **59**, 252–260, 10.1016/j.intimp.2018.03.023 (2018) 10.1016/j.intimp.2018.03.023 29669307

[9] Ma, M. *et al*. Long non-coding RNA nuclear‐enriched abundant transcript 1 inhibition blunts myocardial ischemia reperfusion injury via autophagic flux arrest and apoptosis in streptozotocin-induced diabetic rats. *J. Atheroscler.* **277**, 113–122, 10.1016/j.atherosclerosis.2018.08.031 (2018) 10.1016/j.atherosclerosis.2018.08.031 30205319

[10] Peng, F. Z., Li, M. C., Rong, B. Z., Xiao, L. Y., & Mian, W. The lncRNA Neat1 promotes activation of inflammasomes in macrophages. *J. Nat. Commun.* **10**, 1495, 10.1038/s41467-019-09482-6 (2019) 10.1038/s41467-019-09482-6 PMC6445148 30940803

[11] Dong H.& Yi, D. Z. YY1-induced upregulation of lncRNA NEAT1 contributes to OGD/R injury-induced inflammatory response in cerebral microglial cells via Wnt/β-catenin signaling pathway. *J. In Vitro Cell. Dev. Biol. Anim.* **55**, 501–511. 10.1007/s11626-019-00375-y (2019) 10.1007/s11626-019-00375-y 31286366

[12] Deczkowska, A. *et al*. Disease-associated microglia: A universal immune sensor of neurodegeneration. *J. Cell.* **173,** 1073–1081, 10.1016/j.cell.2018.05.003 (2018) 10.1016/j.cell.2018.05.003 29775591

[13] Ming W. *et al*. Hyperbaric oxygen preconditioning attenuates brain injury after intracerebral hemorrhage by regulating microglia polarization in rats. *J. CNS Neurosci. Ther.* **25**, 1126–1133, 10.1111/cns.13208 (2019) 10.1111/cns.13208 PMC6776759 31411803

[14] Hu, X. *et al*. Microglial and macrophage polarization—New prospects for brain repair. *J. Nat. Rev. Neurol.* **11**, 56–64, 10.1038/nrneurol.2014.207 (2014) 10.1038/nrneurol.2014.207 PMC4395497 25385337

[15] Man, H. L. *et al*. Roles of inflammation response in microglia cell through Toll-like receptors 2/interleukin-23/interleukin-17 pathway in cerebral ischemia/reperfusion injury. *J. Neurosci.* **176**, 162–172, 10.1016/j.neuroscience.2010.11.066 (2010) 10.1016/j.neuroscience.2010.11.066 21182899

[16] Zhang J. *et al*. Altered long non-coding RNA transcriptomic profiles in brain microvascular endothelium after cerebral ischemia. *J. Exp. Neurol.* **277**, 162–170, 10.1016/j.expneurol.2015.12.014 (2015) 10.1016/j.expneurol.2015.12.014 PMC4761283 26746985

[17] Lopez-Neblina, F., Alexander, H. T. & Toledo-Pereyra, L. H. Molecular biology of apoptosis in ischemia and reperfusion. *J. Invest. Surg.* **18**, 335–350, 10.1080/08941930500328862 (2005) 10.1080/08941930500328862 16319055

[18] Ling, Q. *et al*. Roles of the exogenous H2S-mediated SR-A signaling pathway in renal ischemia/reperfusion injury in regulating endoplasmic reticulum stress-induced autophagy in a rat model. *J. Cell Physiol. Biochem.* **41**, 2461–2474, 10.1159/000475915 (2017) 10.1159/000475915 28472786

[19] Zhu, J. *et al.* Ischemic postconditioning-regulated miR-499 protects the rat heart against ischemia/reperfusion injury by inhibiting apoptosis through PDCD4. *J. Cell Physiol. Biochem.* **39**, 2364–2380, 10.1159/000452506 (2016) 10.1159/000452506 27832626

[20] Eefting, F. *et al.* Role of apoptosis in reperfusion injury. *J. Cardiovasc. Res.* **61**, 414–426, 10.1016/j.cardiores.2003.12.023 (2004) 10.1016/j.cardiores.2003.12.023 14962473

[21] William, J. *et al*. Guidelines for the early management of patients with acute ischemic stroke: 2019 update to the 2018 guidelines for the early management of acute ischemic stroke: A guideline for healthcare professionals from the American Heart Association/American Stroke Association. *Stroke*. **50**, e344–e418, https://doi.org/10.1161/STR.0000000000000211 (2019) 10.1161/STR.0000000000000211 31662037

[22] Meng, Y. W. *et al*. Current mechanistic concepts in ischemia and reperfusion injury. *J. Cell. Physiol. Biochem*. **46**, 1650–1667, 10.1159/000489241 (2018) 10.1159/000489241 29694958

[23] Perry, V. H., Nicoll, J. A. R. & Holmes, C. Microglia in neurodegenerative disease. *J. Nat. Rev. Neurol.* **6**, 193–201, 10.1038/nrneurol.2010.17 (2010) 10.1038/nrneurol.2010.17 20234358

[24] Schmid, C. D. *et al.* Differential gene expression in LPS/IFNgamma activated microglia and macrophages: In vitro versus in vivo. *J. Neurochem.* **109**, 117–125, 10.1111/j.1471-4159.2009.05984.x (2009) 10.1111/j.1471-4159.2009.05984.x PMC2766614 19393017

[25] Davalos, D. *et al*. ATP mediates rapid microglial response to local brain injury in vivo. *J. Nat. Neurosci.* **8**, 752–758, 10.1038/nn1472 (2005) 10.1038/nn1472 15895084

[26] Nimmerjahn, A., Kirchhoff, F. & Helmchen, F. Resting microglial cells are highly dynamic surveillants of brain parenchyma in vivo. *J. Sci.* **308**, 1314–1318, 10.1126/science.1110647 (2005) 10.1126/science.1110647 15831717

[27] Lu, H., Shi, Z. W., Hao, L., Zhan, C. D. & Yue, W. Hypoxic preconditioning relieved ischemic cerebral injury by promoting immunomodulation and microglia polarization after middle cerebral artery occlusion in rats. *J. Brain Res.* **1723**, 146338, 10.1016/j.brainres.2019.146388 (2019) 10.1016/j.brainres.2019.146388 31421131

[28] Brendan, D. M. & Cantley, L. C. AKT/PKB signaling: Navigating downstream. *J. Cell.* **129**, 1261–74, 10.1016/j.cell.2007.06.009 (2007) 10.1016/j.cell.2007.06.009 PMC2756685 17604717

[29] Inukai, M. *et al*. Hypoxiamediated cancer stem cells in pseudopalisades with activation of hypoxia-inducible factor-1α/Akt axis in glioblastoma. *J. Hum. Pathol.* **46**, 1496–1505, 10.1016/j.humpath.2015.06.008 (2015) 10.1016/j.humpath.2015.06.008 26256949

[30] Malanga, D. *et al.* The Akt1/IL-6/STAT3 pathway regulates growth of lung tumor initiating cells. *J. Oncotarget.* **6**, 42667–42686, 10.18632/oncotarget.5626 (2015) 10.18632/oncotarget.5626 PMC4767462 26486080

[31] Ruben, O., Christopher, A. M. & Gaylia, J. H. Microglial M1/M2 polarization and metabolic states. *J. Br. J. Pharmacol.* **173**, 649–665, 10.1111/bph.13139 (2016) 10.1111/bph.13139 PMC4742299 25800044

[32] Durafourt, B. A. *et al*. Comparison of polarization properties of human adult microglia and blood-derived macrophages. *J. Glia.* **60**, 717–727, 10.1002/glia.22298 (2012) 10.1002/glia.22298 22290798

