# Supplementary material for: Knockdown lncRNA NEAT1 regulates the activation of microglia and reduces AKT signaling and neuronal apoptosis after cerebral ischemic reperfusion
Source: Sci Rep. 2020 Nov 12;10:19658. doi: 10.1038/s41598-020-71411-1 (PMC7665206; doi:10.1038/s41598-020-71411-1)

Knockdown lncRNA Neat1 regulates the activation of microglia and reduces AKT signaling and neuronal apoptosis after cerebral ischemic reperfusion.

Xunran Ni[1,2*], Qian Su[1*], Wenbo Xia[1,2], Yanli Zhang[1], Kejuan Jia[1], Guozhong Li[1*], Zhiqiang Su[1*]

1. The First Affiliated Hospital of Harbin Medical University, Harbin, PR China.

2. The Key Laboratory of Myocardial Ischemia, Harbin Medical University, Ministry of Education, Harbin, PR China.

Correspondence to zhiqiang Su, The First Affiliated Hospital of Harbin Medical University, 150001, Harbin, PR China. Electronic address: suzhiqiang2020@126.com

Correspondence to guozhong Li, The First Affiliated Hospital of Harbin Medical University, 150001, Harbin, PR China. Electronic address: lgzhyd1962@163.com

* Contributed equally

Supplementary Information

STAT3：

60S:

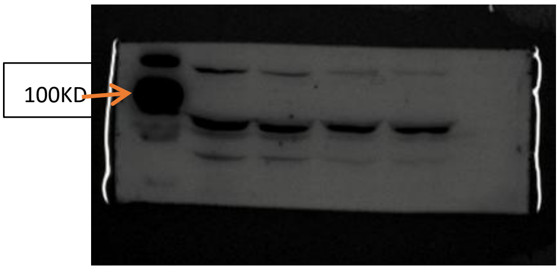

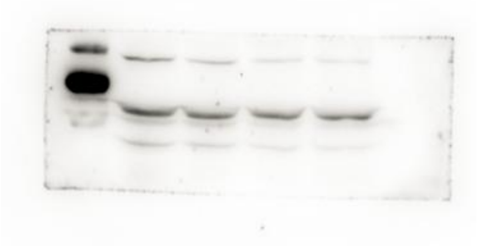

120S：

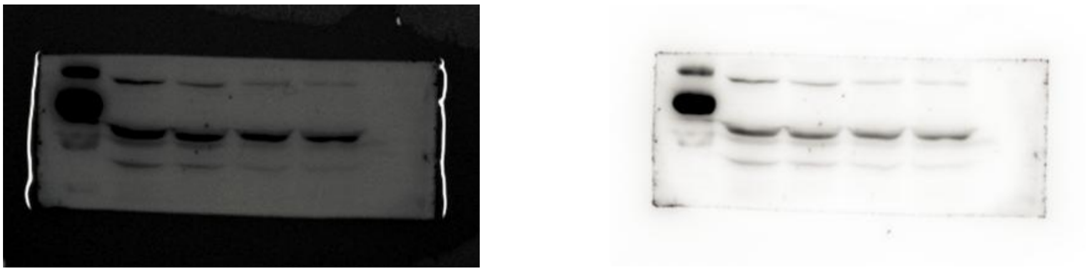

150S:

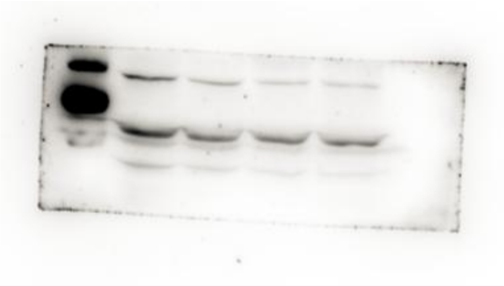

P-STAT3：

3S:

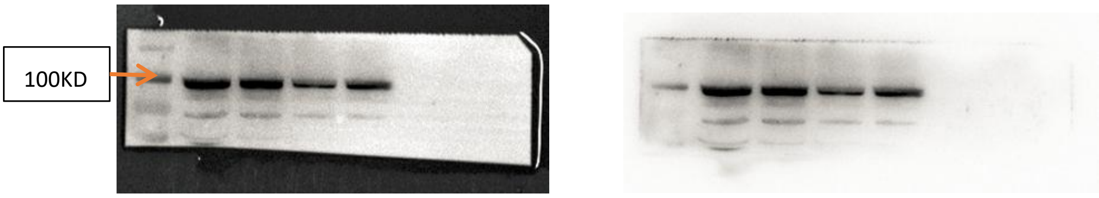

10S:

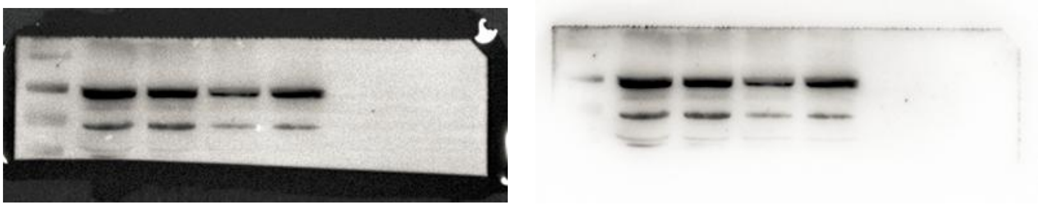

30S:

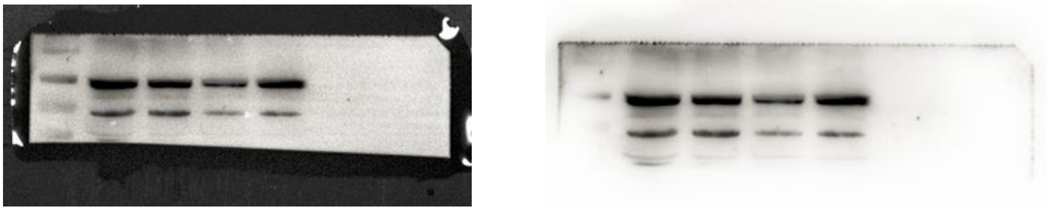

60S:

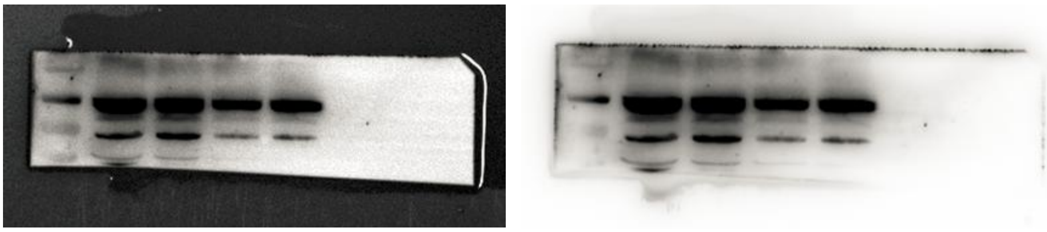

P-AKT:

5S:

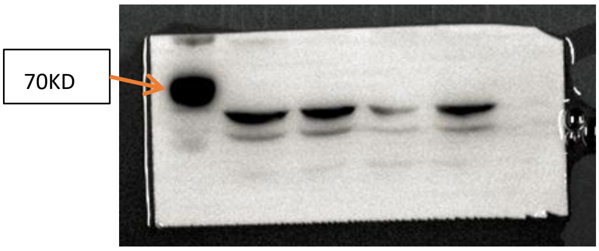

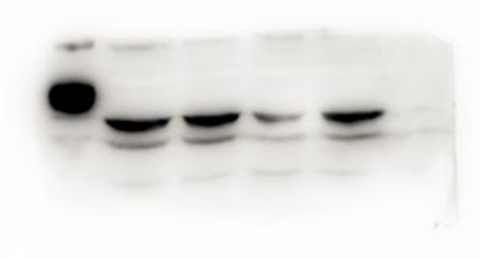

Actin:

10s：

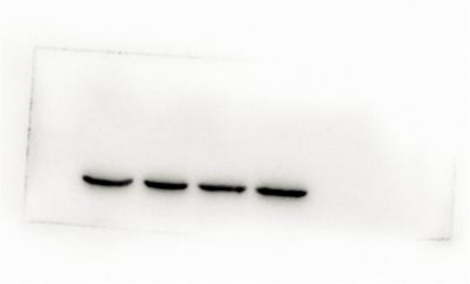

30s：

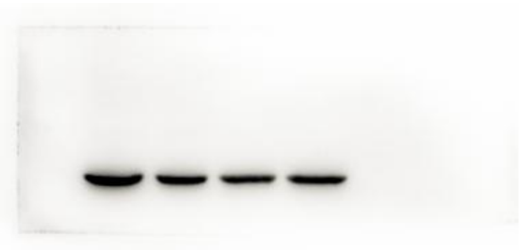

30s:

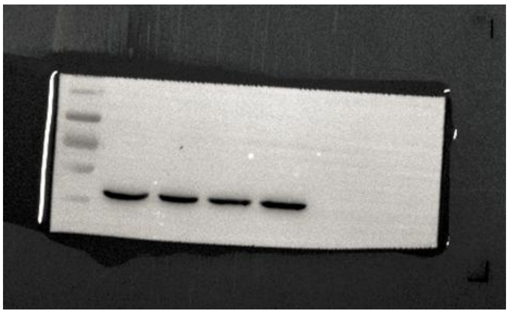

60s:

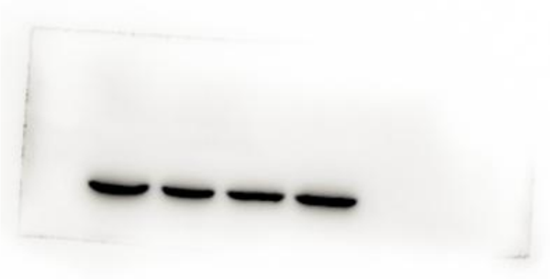

60s:

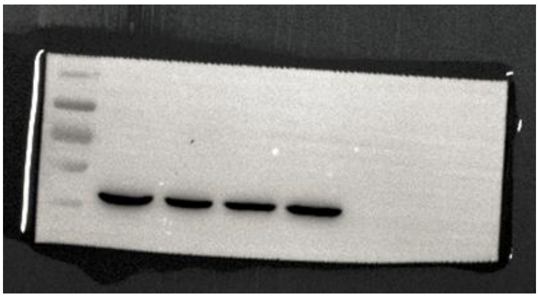

AKT:

10S:

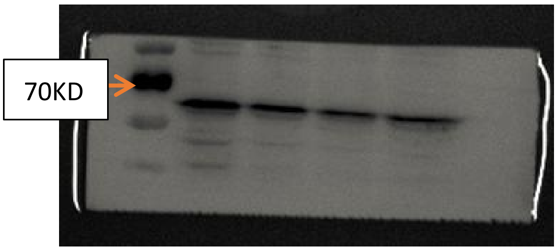

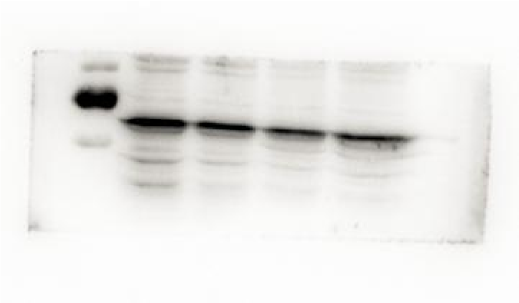

30S:

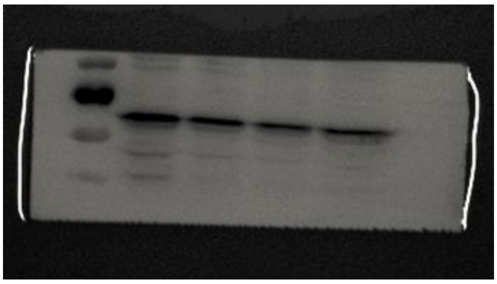

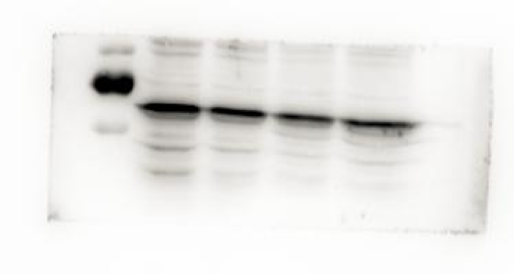

60S:

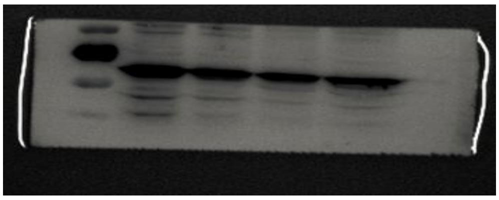

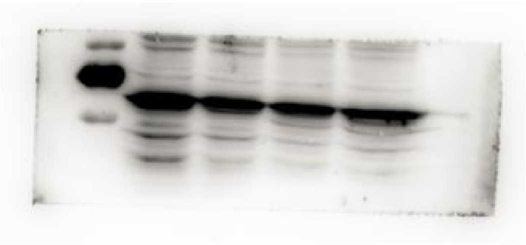

Actin:

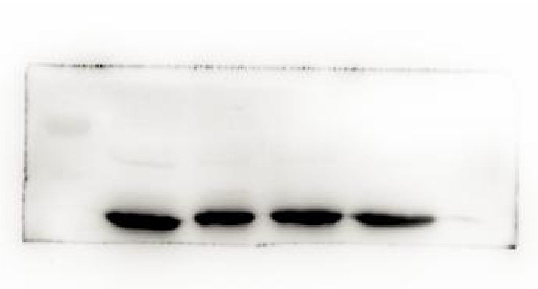

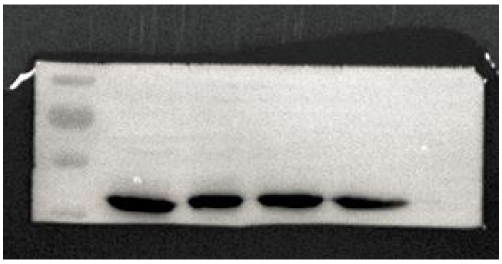

Supplement: Supplementary file 1 — Supplementary Information. [file 41598_2020_71411_MOESM1_ESM.pdf]
